# Clinical utility of circulating tumour cell-based monitoring of late-line chemotherapy for metastatic breast cancer: the randomised CirCe01 trial

**DOI:** 10.1038/s41416-020-01227-3

**Published:** 2021-01-21

**Authors:** Luc Cabel, Frédérique Berger, Paul Cottu, Delphine Loirat, Aurore Rampanou, Etienne Brain, Stacy Cyrille, Hugues Bourgeois, Nicolas Kiavue, Elise Deluche, Sylvain Ladoire, Mario Campone, Jean-Yves Pierga, Francois-Clement Bidard

**Affiliations:** 1 Department of Medical Oncology, Institut Curie, Paris, France; 2 Department of Medical Oncology, Institut Curie, Saint Cloud, France; 3 Circulating Tumor Biomarkers Laboratory, SIRIC2 Institut Curie, Paris, France; 4 UVSQ, Université Paris-Saclay, Saint Cloud, France; 5 Department of Biostatistics, Institut Curie, PSL Research University, Saint Cloud, France; 6 Department of Medical Oncology, Centre Jean Bernard, Le Mans, France; 7 Department of Medical Oncology, CHU de Limoges, Limoges, France; 8 Department of Medical Oncology, CLCC Georges François Leclerc, Dijon, France; 9 Department of Medical Oncology, Institut de cancérologie de l’Ouest, Saint-Herblain, France; 10 Université de Paris, Paris, France

**Keywords:** Breast cancer, Prognostic markers

## Abstract

**Background:**

CirCe01 trial aimed to assess the clinical utility of circulating tumour cell (CTC)-based monitoring in metastatic breast cancer (MBC) patients beyond the third line of chemotherapy (LC).

**Methods:**

CirCe01 was a prospective, multicentre, randomised trial (NCT01349842) that included patients with MBC after two systemic LC. Patients with ≥5 CTC/7.5 mL (CellSearch®) were randomised between the CTC-driven and the standard arm. In the CTC arm, changes in CTC count were assessed at the first cycle of each LC; patients in whom CTC levels predicted early tumour progression had to switch to a subsequent LC.

**Results:**

Greater than or equal to 5 CTC/7.5 mL were observed in *N* = 101/204 patients. In the CTC arm (*N* = 51), 43 (83%) and 18 (44%) patients completed CTC monitoring in the third and fourth lines, respectively, and 18 (42%) and 11 (61%) of these patients, respectively, had no CTC response. Thirteen (72%) and 5 (46%) of these patients underwent early switch to the next LC. Overall survival was not different between the two arms (hazard ratio = 0.95, 95% confidence interval = [0.6;1.4], *p* = 0.8). In subgroup analyses, patients with no CTC response who switched chemotherapy experienced longer survival than patients who did not.

**Conclusions:**

Due to the limited accrual and compliance, this trial failed to demonstrate the clinical utility of CTC monitoring.

**Clinical Trial Registration:**

NCT, NCT01349842, https://clinicaltrials.gov/ct2/show/NCT01349842, registered 9 May 2011.

## Background

Metastatic breast cancer (MBC) is the leading cause of cancer death among women worldwide.^1^ As stated by the Advanced Breast Cancer consensus conference, MBC is an incurable but treatable disease, with a median overall survival >5 years in the HER2-positive (HER2+) and oestrogen receptor-positive (ER+) subgroups.^2^ While HER2+ and triple-negative MBC patients are treated with frontline chemotherapy, ER+ HER2− MBC patients are treated with first-line endocrine therapy, possibly followed by chemotherapy after the onset of overt endocrine resistance.^3^ While the use of eribulin as second- or third-line chemotherapy has demonstrated a statistically significant benefit on overall survival,^4^ the survival benefit of subsequent late lines of chemotherapy remains a subject of debate. No clinical characteristics or markers are currently available to identify a population of patients that would benefit from administration of late-line chemotherapy.^5–8^

Circulating tumour cells (CTCs) are rare tumour cells that can be detected in peripheral blood.^9^ In a first study on 177 MBC patients published in 2004,^10^ ≥5 CTC/7.5 mL was proposed as a cut-off to distinguish MBC patients with good vs. poor survival at baseline. Further analyses showed that CTC changes 3–5 weeks after the initiation of a new line of chemotherapy, using the same cut-off, were also associated with treatment efficacy.^11^ Based on these clinical validity data, the CellSearch® system was approved by the US Food and Drug Administration as an aid to the monitoring of patients with MBC. The early change of CTC count was then confirmed as a prognostic marker by other studies worldwide and eventually became a level-of-evidence 1 prognostic biomarker following the European Pooled Analysis of CTC (EPAC^12^) A combination of EPAC and American data further substantiated the clinical validity of CTC count in MBC.^13^

The key issue then became a demonstration of the clinical utility of CTC-based monitoring, that is, taking early CTC count changes into account would improve the MBC patient’s clinical outcome. We report here the results of CirCe01, a CTC-based prospective trial conducted in the late-line setting (i.e. third line and beyond), in which CTC monitoring had to be repeated at each subsequent line of chemotherapy (starting from the third line), in order to evaluate whether repeated CTC-guided changes of chemotherapy can provide a significant survival benefit.

## Methods

### Ethics

This prospective, multicentre, open-label, randomised trial (six centres) was approved by the regional ethics board (approved by “Comité de Protection des Personnes—Ile de France”) and identified as NCT01349842 (registered 9 May 2011). A written informed consent was obtained from all participants.

### Trial design and procedures

Inclusion criteria were: women aged 18 years or older with MBC, with any performance status (PS), who progressed after two lines of systemic chemotherapy administered for metastatic disease and for whom a third line of chemotherapy was considered.

**Fig. 1:**
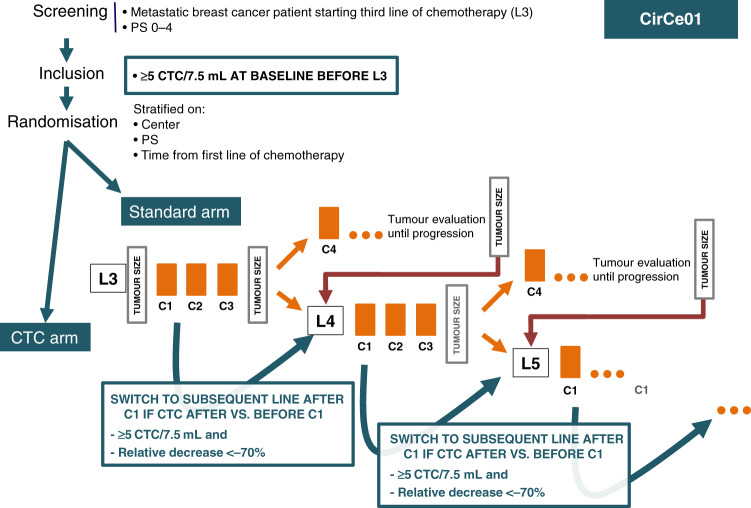
Design of the CirCe01 trial. CTC circulating tumour cells, L line of systemic chemotherapy, PS performance status.

The trial design is displayed in Fig. 1. The protocol did not specify which chemotherapy regimens were to be used, as treatments were discussed at team meetings on an individual basis. Using the CellSearch® system, a first CTC count was obtained prior to the first cycle of third-line chemotherapy (C1L3). Patients with <5 CTC/7.5 mL at inclusion were considered to be non-evaluable for CTC count and were not randomised. Imaging was performed every three cycles to capture their third-line progression-free survival (PFS); overall survival (OS) was also prospectively collected.

Patients with ≥5 CTC/7.5 mL at inclusion were randomised 1:1 between the CTC arm (arm A) and the standard arm (arm B), using sealed envelopes and permutation blocks (with a block size of six). Randomisation was performed centrally (at Institut Curie) and stratified by PS (0–1 vs. 2–3) and time between diagnosis of metastatic disease and inclusion in the study (≤15 vs. >15 months). CTC count was not repeated in the standard arm and patients were treated according to tumour imaging, performed every three cycles. Chemotherapy change was allowed upon disease progression, with no limitation of the number of treatment lines. Third-line PFS and OS were prospectively registered.

In the CTC arm, CTC count was repeated at day 14 to have a CTC count result prior to the second cycle of third-line chemotherapy. A ≥70% decrease of the baseline CTC count or a fall to below <5 CTC/7.5 mL was considered to be indicative of a potential response to chemotherapy. This composite endpoint was established in the previously reported run-in step,^14^ in which it was shown to have a better clinical validity than the standard <5 CTC/7.5 mL cut-off. Patients with a CTC count meeting the predefined CTC response criteria were maintained on the same chemotherapy regimen until disease progression based on radiological evaluation (RECIST (Response Evaluation Criteria in Solid Tumours)). Patients predicted to experience early tumour progression according to CTC monitoring (i.e. no CTC response according to the above-mentioned composite endpoint) had to discontinue their third-line chemotherapy and start a fourth-line chemotherapy. Importantly, this CTC-based monitoring had to be repeated for each new line of chemotherapy in all patients randomised to the CTC arm, except when the patient was eligible and included in another clinical trial during subsequent lines of treatment. This implied, for example, that a patient randomised to the CTC arm could receive three different lines of chemotherapy over a 9-week period when none of these lines induced a CTC response. Clinical and radiological evaluations were performed at least every three cycles in the CTC arm, as in the standard arm.

### Statistics

The primary endpoint was OS from randomisation, and 190 events were required (assumed hazard ratio (HR) = 0.66, two-sided type I error of 5%, power of 80%). Considering a CTC ≥ 5 CTC/7.5 mL detection rate of 50% and assuming 10% CTC detection failure, it was initially planned to screen 669 patients.

The primary efficacy analysis was performed for patients randomly assigned to one of the two interventional arms (i.e. intention-to-treat analysis). Categorical variables were compared by *χ*^2^ or Fisher’s exact tests, and continuous variables were compared by Student’s *t* test or Wilcoxon’s rank-sum test. OS was defined as the time from randomisation to death from any cause. PFS was defined as the time from randomisation to tumour progression or death from any cause, whichever came first. Patients with no events were censored at the date of their last visit. OS and PFS functions were computed using the Kaplan–Meier method. HRs of arm A vs. arm B and their 95% confidence intervals (95% CIs) were estimated using a Cox proportional hazards model for OS and PFS and a log-rank test was used to compare treatment arms. OS and PFS of patients of arm A with CTC response or with no CTC response who followed the mandatory switch were assessed in post hoc analyses. Statistical analyses were performed with R software (version 3.4.2). A *p* value < 0.05 was considered to be statistically significant.

## Results

The study was initiated in March 2012 and 207 patients were included up until October 2015, when accrual was terminated by the study steering committee. This decision was based on both the slow accrual and frequent non-compliance with the CTC monitoring-based changes of chemotherapy.

**Fig. 2:**
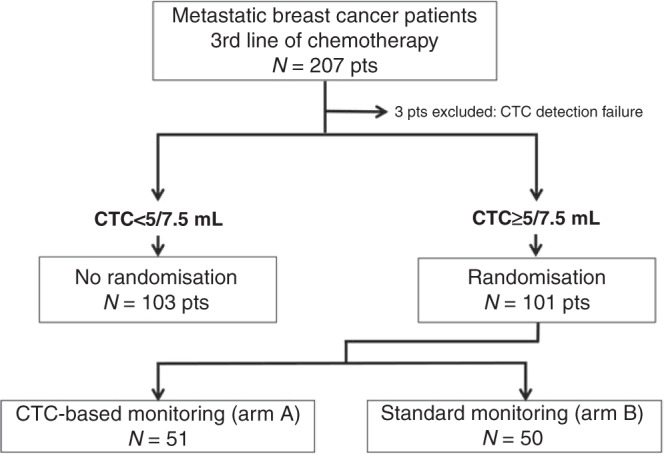
Study flow chart. CTC: circulating tumor cells.

The study flow chart is displayed in Fig. 2. Baseline CTC count was obtained in 204 patients, with a median count of 4 CTC/7.5 mL (range: 0–74,775, interquartile range (IQR) = 1–34). One hundred and six patients were not randomised because of a baseline CTC count <5 CTC/7.5 mL. One hundred and one patients with ≥5 CTC/7.5 mL at baseline were randomised between CTC-based (arm A, *N* = 51 patients) and standard (arm B, *N* = 50 patients) monitoring. Among the randomised patients (*N* = 101), 80% had a PFS >6 months in the first line of chemotherapy (median PFS 11.9 months, IQR = 7.3–24.4) and 58% in the second line of chemotherapy in the metastatic setting (median PFS 7.3 months, IQR 3.8–13.6).

### Patient characteristics

Patient characteristics and associated CTC counts are displayed in Table 1, and chemotherapies received from lined 1 to 4 are displayed in Supplemental Table 1.

**Table 1:** Patient characteristics.

	<5 CTC/7.5 mL Not randomised *N* = 106	≥5 CTC/7.5 mL Arm A CTC-based monitoring *N* = 51	≥5 CTC/7.5 mL Arm B standard monitoring *N* = 50	*P* value <5 CTC vs. ≥5 CTC/7.5 mL	*P* value Arm A vs. arm B
Median age [IQR]	59 [51–66]	59 [52–65]	60 [51–65]	0.36	0.43
Menopausal status					
Premenopausal	33 (34%)	16 (32%)	13 (27%)	0.61	0.75
Postmenopausal	64 (66%)	34 (68%)	35 (73%)		
NA	9	1	2		
Performance status					
0–1	106	40 (87%)	41 (85%)	NA	0.99
2–4		6 (13%)	7 (15%)		
NA		5	2		
Histology					
IC-NST	97 (92.4%)	36 (71%)	42 (84%)	0.004	0.15
Lobular	8 (7.6%)	15 (29%)	8 (16%)		
Primary tumour grade					
1	13 (13%)	4 (8%)	5 (10%)	0.69	0.90
2	51 (52%)	26 (53%)	26 (54%)		
3	35 (35%)	19 (39%)	17 (36%)		
NA	7	2	2		
Primary tumour subtype					
Triple negative	15 (14%)	7 (14%)	4 (8%)	0.03	0.55
HR+ HER2−	85 (80%)	44 (86%)	46 (92%)		
HER2+	6 (6%)	0	0		
Number of metastatic sites (at relapse)					
<3	75 (72%)	32 (64%)	27 (54%)	0.07	0.42
≥3	29 (28%)	18 (36%)	23 (46%)		
NA	2	1	0		
LDH					
Normal	106	9 (23%)	11 (26%)	NA	0.79
>ULN		30 (77%)	32 (74%)		
NA		12	7		
Median baseline CTC level [IQR]	1 [0–2]	40 [15–88]	33 [11–105]	<0.001	0.78

*HER2+* HER2 positive, *HR* hormone receptor, *IC-NST* invasive carcinoma of no specific type, *ULN* upper limit of normal, *NA* data not available.

A baseline, CTC count ≥5 CTC/7.5 mL was associated with tumour histological type, molecular subtype and, marginally, the number of metastatic sites at the time of metastatic relapse. No significant difference in terms of patient characteristics was observed between the two arms of the randomised population (with ≥5 CTC/7.5 mL). At baseline, CTC level was a prognostic factor in univariate analysis in the overall study population patients; patients with <5 and ≥5 CTC/7.5 mL at inclusion had a median PFS of 12.2 months 95% CI [10.2;14.4] and 3.6 months 95% CI [3.3;4.9] (HR = 3.1; 95% CI [2.3;4.1], *p* < 0.001), and an OS of 19.4 months 95%CI [16.7;26.3] and 10.2 months 95% CI [8.1;13.9] (HR = 2.3; 95% CI = [1.7;3.0], *p* < 0.001), respectively (Additional Data 1 and Fig. S1).

### Treatment received and outcome

The overall median time on chemotherapy during this study was not statistically different between arm A and arm B (median of 7.4 months IQR [2.5–16.7] vs. median of 8.3 months IQR [4.7–14.7], *p* = 0.81). At the time of analysis, the median follow-up for randomised patients was 62 months (range: 9–81) with 100 events for PFS and OS (>99% maturity).

**Fig. 3:**
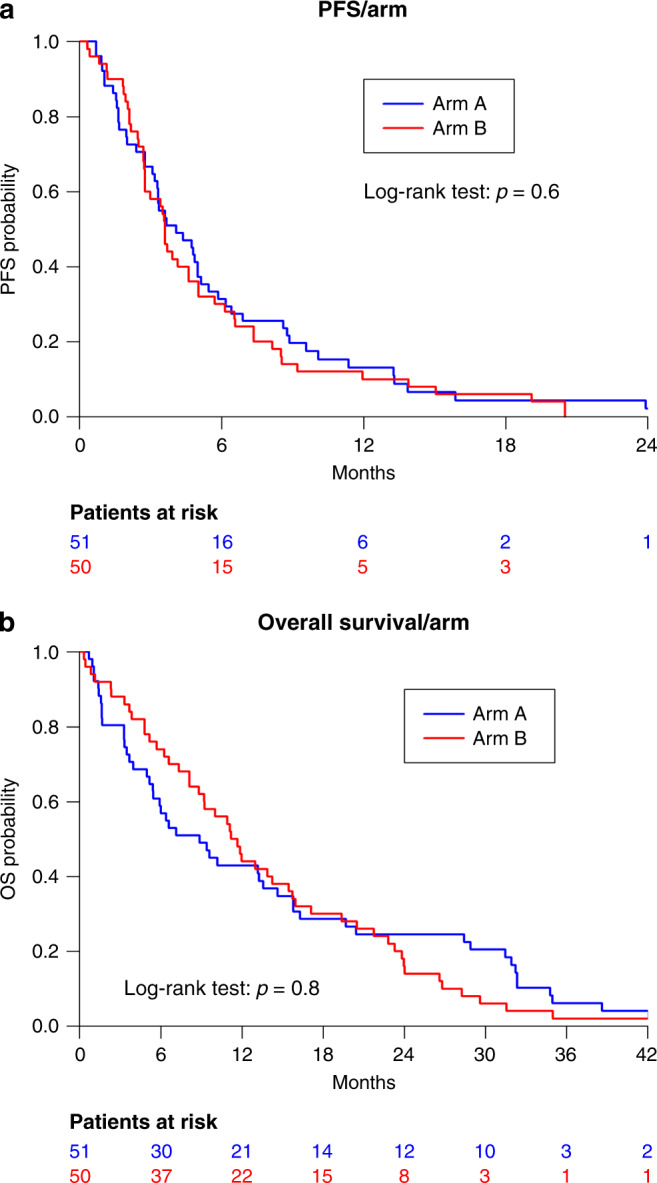
Survival. Progression-free survival (**a**) and overall survival (**b**) according to the study arm. Arm A CTC-based monitoring, Arm B standard monitoring.

In patients allocated to the early CTC-based monitoring (arm A), the median PFS was 4.1 months (95% CI = [3.3;5.5]) and the median OS was 8.9 months (95%CI = [5.5;15.8]). In the standard arm (arm B), the median PFS was 3.6 months (95% CI = [2.8;5.0]) and the median OS was 11.4 months (95% CI = [8.8;16.0]). Median PFS and OS were not statistically different between arms A and B, with an HR of 0.9 (95% CI = [0.6;1.3], *p* = 0.6) and 0.95 (95% CI = [0.6;1.4], *p* = 0.8), respectively (Fig. 3). Other prognostic factors for PFS or OS are displayed in Supplemental Table 2. ER+-HER2− status was significantly associated with a longer PFS (HR = 0.45, 95% CI = [0.24;0.86], *p* = 0.03); a similar trend was observed with OS, although not statistically significant (HR = 0.55, 95% CI = [0.29;0.1.03], *p* = 0.08). The presence of three or more metastatic sites at inclusion was found to be associated with a shorter OS (HR = 1.57, 95% CI = [1.04;2.4], *p* = 0.03), but had no impact on PFS (HR = 0.94, 95% CI = [0.6;1.4], *p* = 0.77).

### Compliance with the protocol and outcome by a line of therapy

**Fig. 4:**
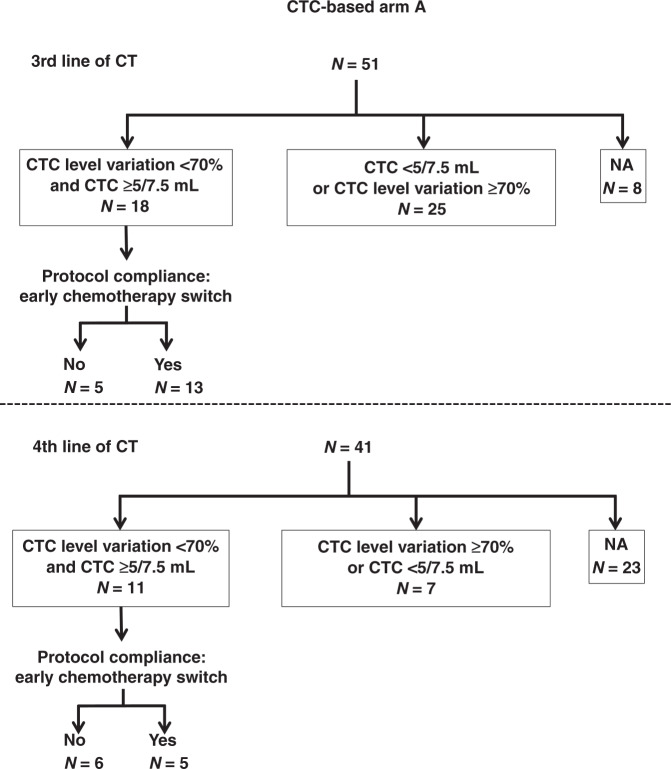
CTC level variation and protocol compliance at the third and fourth lines of chemotherapy. CTC circulating tumour cell, CT chemotherapy.

The availability of early CTC monitoring and CTC results among patients randomised to arm A are displayed in Fig. 4.

Among the 51 patients in arm A receiving third-line chemotherapy and attending a hospital visit at 2 weeks for the second blood draw, a second CTC count was obtained in 43 patients (84%). CTC monitoring showed a CTC response (≥70% relative reduction or <5 CTC/7.5 mL absolute count at week 2) in 25 patients (58%), who were encouraged to continue their third-line chemotherapy (Fig. 4), and these patients experienced a longer OS (*p* < 0.001) (Additional Data 1 and Fig. S2). The median PFS in these 25 patients with a CTC response was 5.5 months 95% CI = [3.3;8.8] (Table 2) and their median OS was 16.3 months (95% CI = [13.6;31.9]). In contrast, 18 patients (42%) did not display any CTC response to third-line chemotherapy. Only 13 of these 18 patients complied with the mandatory switch to fourth-line chemotherapy, as defined in the study protocol. In these 13 patients, the median PFS was 4.4 months (95% CI = [1.6;NA]), while the median OS was 6.0 months 95% CI [3.3;NA] (Additional Data 1 and Fig. S3). In the remaining five patients, third-line chemotherapy was continued despite the absence of CTC response. These five patients experienced a median PFS of 3.3 months (95% CI = [2.4;NA]) and a median OS of 3.7 months (95% CI = [3.3;NA]), and chemotherapies received are described in Supplemental Table 3.

**Table 2:** Outcome of patients in whom CTC changes were assessed, according to the line of chemotherapy and compliance with the protocol.

	Number of patients	PFS [95% CI]	OS [95% CI]
Third line			
Low baseline CTC	103	12.2 [10.2–14.4]	19.4 [16.7–26.3]
High baseline CTC - CTC response	25	5.5 [3.3–8.8]	16.3 [13.6–31.9]
High baseline CTC - No CTC response - Chemotherapy switch	13	4.4 [1.6–NA]	6.0 [3.3–NA]
High baseline CTC - No CTC response - No chemotherapy switch	5	3.3 [2.4–NA]	3.7 [3.3–NA]
Fourth line			
High baseline CTC - CTC response	7	11.6 [7.4–NA]	15.8 [8.9–NA]
High baseline CTC - No CTC response - Chemotherapy switch	5	11.0 [8.0–NA]	31.9 [19.7–NA]
High baseline CTC - No CTC response - No chemotherapy switch	6	4.0 [3.4–NA]	6.0 [5.0–NA]

Due to the small number of patients, no comparative statistical analysis was performed.

Fourth-line chemotherapy was administered to 41 of the 51 patients randomised to arm A. CTC monitoring of fourth-line chemotherapy was available for 18 patients (44%). These 18 patients had a median PFS of 2 months (range 0.6–17) at third-line chemotherapy. Seven (39%) patients displayed a CTC response, and experienced a median PFS and OS of 11.6 months (95% CI = [7.4;NA]) and 15.8 months (95% CI = [8.9;NA]), respectively. Eleven (61%) patients had no CTC response, which should have triggered an early switch to another line of chemotherapy. This switch was performed in five (46%) of these 11 patients; these five patients had a median PFS and OS of 11.1 months (95% CI = [8.0;NA]) and 31.9 months (95% CI = [19.7;NA]), respectively. The remaining six patients, who continued with their fourth-line chemotherapy despite the absence of CTC response, had a median PFS and OS of 4 months (95% CI = [3.4;NA]) and 6 months (95% CI = [5;NA]), respectively. PFS and OS results according to the line of treatment and compliance with the protocol are displayed in Fig. 4.

Twenty-seven patients received fifth-line chemotherapy and 15 patients received sixth-line chemotherapy, but CTC monitoring was obtained for only a small number of patients (data not shown).

## Discussion

CirCe01 is the second trial to address the clinical utility of monitoring chemotherapy by early CTC changes in MBC patients. The first trial, SWOG S0500, selected 120 MBC patients with no CTC response (according to the standard ≥5 CTC/7.5 mL cut-off) to first-line chemotherapy and compared continuation of first-line chemotherapy (until disease progression) vs. early initiation of second-line chemotherapy.^15,16^ This trial failed to demonstrate any utility of CTC count;^15^ the potential reasons for the failure of this trial have been discussed elsewhere,^17^ including the fact that the study population (MBC exhibiting spontaneous, immediate resistance to first-line chemotherapy) was likely refractory to all forms of chemotherapy; the ≥5 CTC/7.5 mL cut-off was also not initially optimised to “predict” the efficacy of chemotherapy. The CirCe01 trial consequently adopted a different design: (i) the third-line setting was explored to avoid the selection of de novo chemo-resistant tumours; (ii) a modified cut-off to call a CTC response was defined in an observational, run-in phase of the study.^14^ In the run-in phase of the study, 87% of patients with no CTC response had a PFS <4 months, while 70% of patients with a CTC response had a PFS >4 months; (iii) the CTC test was repeated at each new line of chemotherapy in order to select the most appropriate chemotherapy option.^14^

Despite these theoretical considerations, CirCe01 did not achieve its main objective, as patients in the CTC-based monitoring arm did not have a longer OS. This trial was marked by poor acceptance of the CTC-based treatment switch and possibly insufficient accrual. While the main study result was negative (considering the overall study population), compliance with the proposed early switch of chemotherapy in patients with no CTC response nevertheless resulted in quantitatively longer median PFS and OS.

Poor compliance with the study protocol markedly decreased the power of CirCe01, as many patients in the CTC arm were actually managed by standard monitoring. The reasons for these unexpected protocol deviations were not fully captured in the study case report forms. For logistical reasons, several patients did not undergo the second blood draw that had to be performed at their hospital, which was required for monitoring of CTC changes. CTC response could therefore not be assessed in these patients, who were then off-study (i.e. CTC monitoring was not proposed at subsequent lines of therapy). Retrospective visual inspection of the patient charts also showed that some patients experienced improvement of clinical or laboratory parameters that—according to the patient and/or the investigator—justified dismissing the absence of CTC response. Instead of switching to a subsequent line of chemotherapy, these patients continued with the same chemotherapy and experienced the shortest survival of all patient subgroups, in both third and fourth lines, suggesting that the absence of CTC response is a marker that must be taken into account. These non-protocol compliant patients were also considered to be off-study, further reducing the actual number of patients with CTC monitoring among the patients randomised in the CTC-driven arm and, in turn, the study power.

## Conclusion

Early CTC-based monitoring of the efficacy of third-line and beyond chemotherapy for MBC failed to improve patient survival. The high rate of non-compliance with the study protocol highlights that early results suggesting poor prognosis are often dismissed when no other recognised alternative treatment option is available.

## Supplementary information

Supplemental table and Fig

## Data Availability

The data that support the findings of this study are available from Institut Curie, but restrictions apply to the availability of these data, which were used under license for the current study, and so are not publicly available. Data are however available from the authors upon reasonable request and with permission of Institut Curie.
