# Supplementary material for: Clinical utility of circulating tumour cell-based monitoring of late-line chemotherapy for metastatic breast cancer: the randomised CirCe01 trial
Source: Br J Cancer. 2021 Jan 21;124(7):1207–13. doi: 10.1038/s41416-020-01227-3 (PMC8007590; doi:10.1038/s41416-020-01227-3)
Supplement: Supplementary file 1 — Supplemental table and Fig [file 41416_2020_1227_MOESM1_ESM.docx]

| **Treatment** | **L1** | | **L2** | | **L3** | | **L4** | |
| --- | --- | --- | --- | --- | --- | --- | --- | --- |
|  | Arm A  N=51 | Arm B  N=50 | Arm A  N=51 | Arm B  N=50 | Arm A  N=51 | Arm B  N=50 | Arm A  N=41 | Arm B  N=39 |
| **Anthracyclin-based** | 7 | 5 | 5 | 6 | 9 | 11 | 3 | 4 |
| **Taxane** | 31 | 33 | 10 | 13 | 10 | 10 | 4 | 1 |
| **Capecitabine / 5FU** | 16 | 17 | 27 | 26 | 5 | 7 | 2 | 4 |
| **Navelbine** | 0 | 0 | 2 | 4 | 8 | 10 | 5 | 5 |
| **Gemcitabine** | 0 | 0 | 1 | 0 | 2 | 3 | 7 | 3 |
| **Eribulin** | 0 | 0 | 1 | 2 | 14 | 5 | 12 | 14 |
| **Carboplatin** | 1 | 0 | 1 | 0 | 0 | 0 | 1 | 0 |
| **Bevacizumab** | 18 | 20 | 1 | 0 | 0 | 0 | 0 | 0 |
| **Other** | 0 | 0 | 3 | 1 | 3 | 4 | 7 | 8 |

**Supplemental Table 1: Chemotherapy regimen received in line (L) 1 to 4.**

Total may be greater than the number of patients because several drugs may have been administered in one line.

| **Progression-Free Survival** | | | | |
| --- | --- | --- | --- | --- |
|  | **N** | **HR** | **95%CI(HR)** | **P-value** |
| **Age** | 101 | 1.14 | [0.76 ; 1.7] | 0.52 |
| **Baseline CTC/7.5mL** |  |  |  |  |
| <35 | 50 | 1 |  | 0.11 |
| ≥35 | 51 | 1.38 | [0.93 ; 2.05] |  |
| **Tumor subtype** |  |  |  |  |
| Triple-Negative | 11 | 1 |  | **0.027** |
| HR+ HER2- | 90 | 0.45 | [0.24 ; 0.86] |  |
| **Performance status** |  |  |  |  |
| PS 0-1 | 81 | 1 |  | 0.20 |
| PS 2-4 | 13 | 1.5 | [0.83 ; 2.71] |  |
| **Number of metastatic sites** |  |  |  |  |
| <3 | 56 | 1 |  | 0.77 |
| ≥3 | 45 | 0.94 | [0.63 ; 1.4] |  |
| **LDH** |  |  |  |  |
| LDH Normal | 20 | 1 |  | 0.17 |
| LDH > ULN | 62 | 1.41 | [0.84 ; 2.37] |  |

| **Overall Survival** | | | | |
| --- | --- | --- | --- | --- |
|  | **N** | **HR** | **95%CI(HR)** | **P-value** |
| **Age** | 101 | 1.28 | [0.86 ; 1.91] | 0.23 |
| **Baseline CTC/7.5mL** |  |  |  |  |
| <35 | 50 | 1 |  | 0.20 |
| ≥35 | 51 | 1.29 | [0.87 ; 1.93] |  |
| **Tumor subtype** |  |  |  |  |
| Triple-Negative | 11 | 1 |  | 0.084 |
| HR+ HER2- | 90 | 0.55 | [0.29 ; 1.03] |  |
| **Performance status** |  |  |  |  |
| PS 0-1 | 81 | 1 |  | 0.065 |
| PS 2-4 | 13 | 1.82 | [1.01 ; 3.29] |  |
| **Number of metastatic sites** |  |  |  |  |
| <3 | 56 | 1 |  | **0.033** |
| ≥3 | 45 | 1.57 | [1.04 ; 2.38] |  |
| **LDH** |  |  |  |  |
| LDH Normal | 20 | 1 |  | 0.16 |
| LDH > ULN | 62 | 1.43 | [0.86 ; 2.38] |  |

**Supplemental Table 2: Prognostic factors of Progression-free survival and Overall survival in univariate analysis.**

For CTC analysis, 35 CTC/7.5ml is the median in this population

HR: Hazard-ratio, HER2+: HER2-positive, HR+: hormone receptor positive, ULN: upper limit of normal

| **Patient** | **L3C1** | **L4C1** | **Patient** | **L4C1** | **L5C1** |
| --- | --- | --- | --- | --- | --- |
| 1 | Paclitaxel | Vinorelbine | 1 | Paclitaxel | Vinorelbine |
| 2 | Eribulin | * | 2 | Paclitaxel | Vinorelbine |
| 3 | Capecitabine | * | 3 | Vinorelbine | Gemcitabine |
| 4 | Capecitabine | Eribulin | 4 | Eribulin | Paclitaxel |
| 5 | Eribulin | Gemcitabine | 5 | Capecitabine | Vinorelbine |
| 6 | Doxorubicin | Eribulin |  |  |  |
| 7 | Paclitaxel | Vinorelbine |  |  |  |
| 8 | Cyclophosphamide + Doxorubicin | Gemcitabine |  |  |  |
| 9 | Cyclophosphamide + Doxorubicin | Eribulin |  |  |  |
| 10 | Cyclophosphamide | Gemcitabine |  |  |  |
| 11 | Eribulin | Paclitaxel |  |  |  |
| 12 | Eribulin | Paclitaxel |  |  |  |
| 13 | Liposomal Doxorubicin | Gemcitabine |  |  |  |

**Supplemental Table 3: Early chemotherapy switch in L3 (n=13) and in L4 (n=5)**

* Switch planned but patient died before the new line

**Figure S1: Overall survival (A) and Progression-free survival (B) according to baseline CTC level ≥5 or <5**


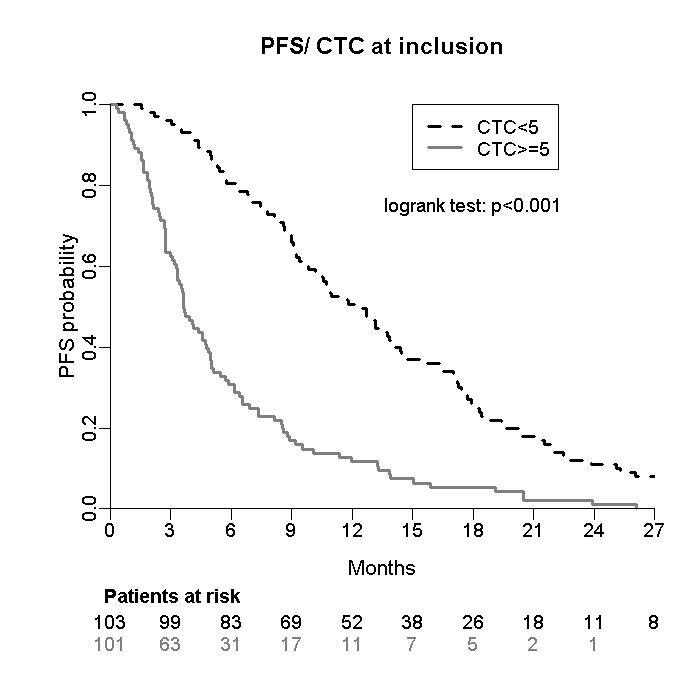

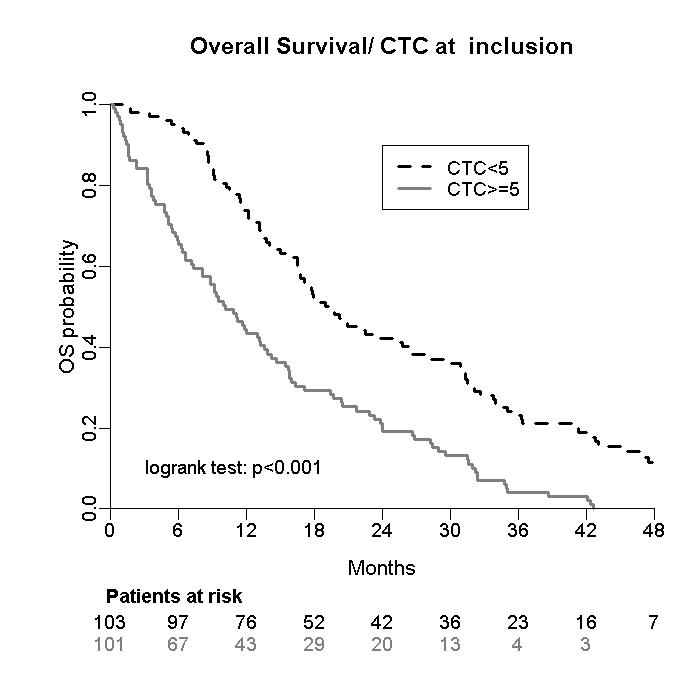


**Figure S2: Progression-free survival (A) and Overall survival (B) according to baseline CTC level and CTC reduction at third line of chemotherapy**

**
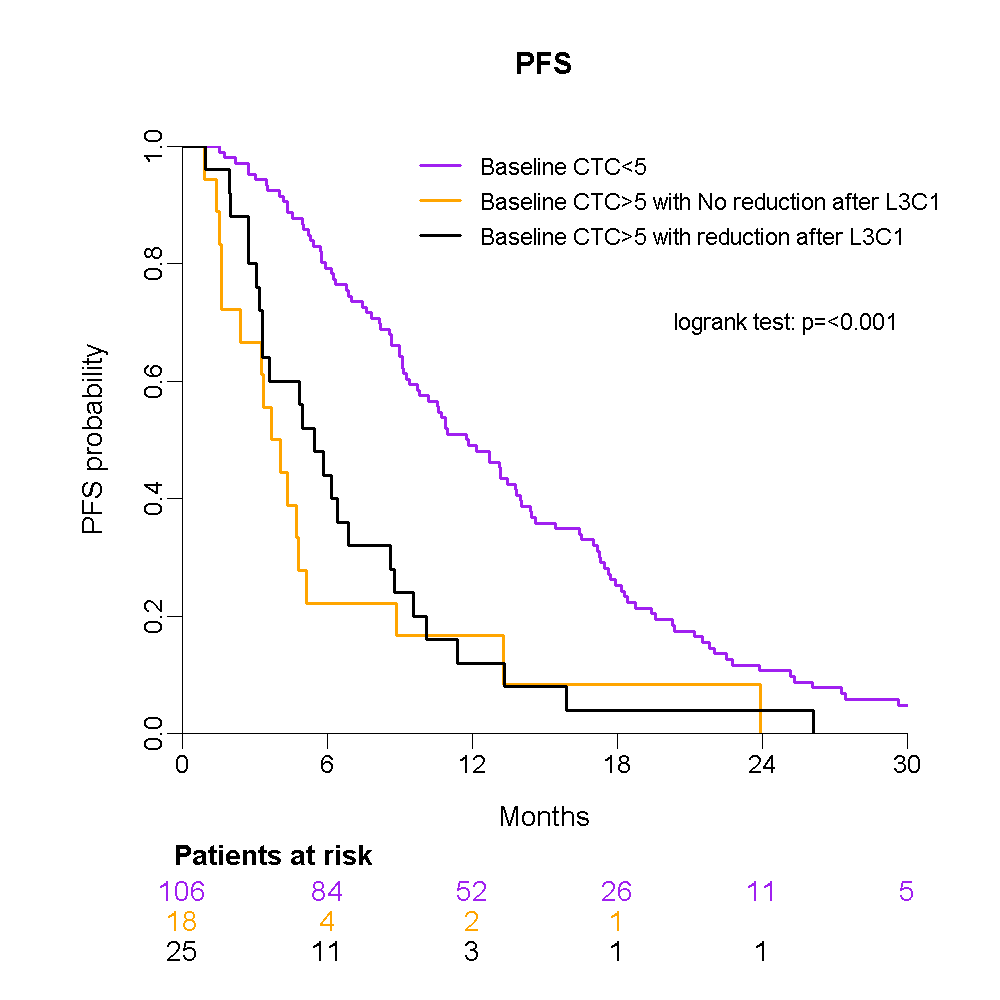

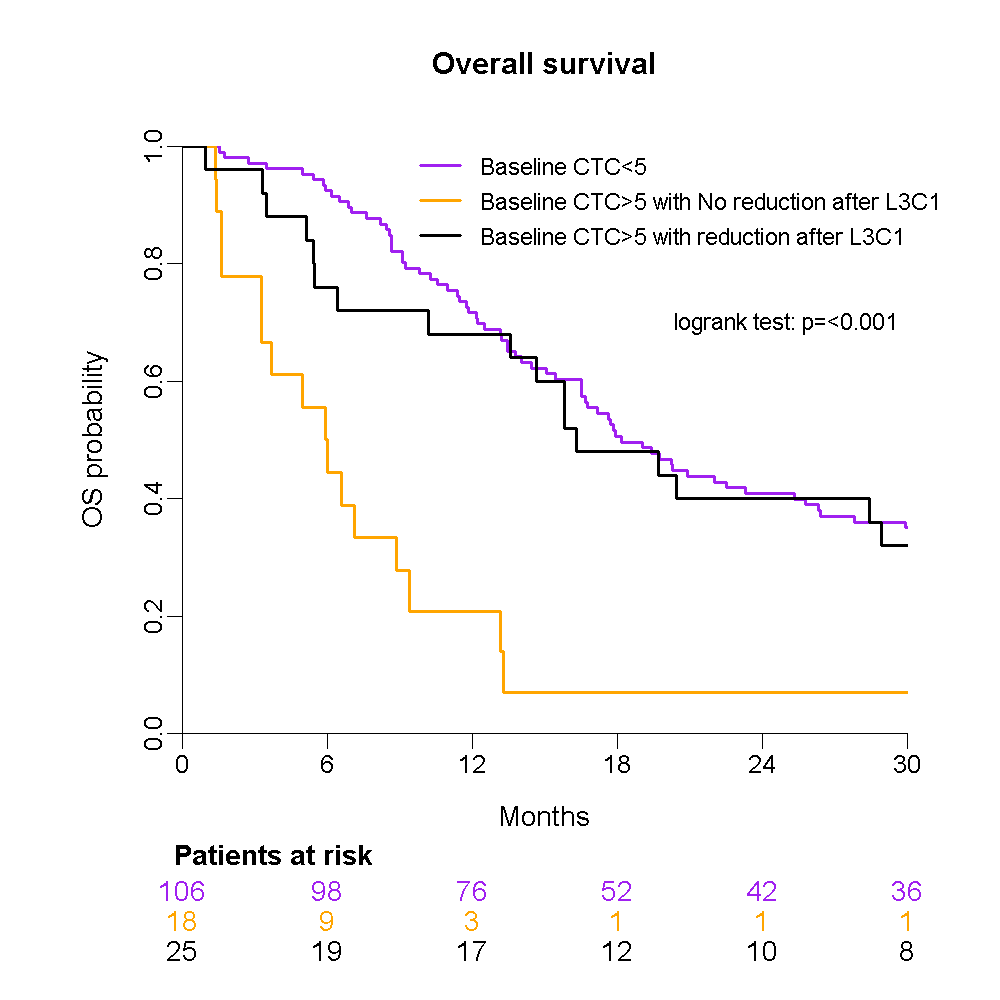
**

**B**

**A**

**Figure S3: Exploratory analysis in Arm A (CTC-based strategy) of patients who effectively had an early chemotherapy switch (protocol compliance: YES) versus others (protocol compliance: NO), at 3rd and 4th lines of chemotherapy for progression-free survival (PFS) and overall survival (OS).**


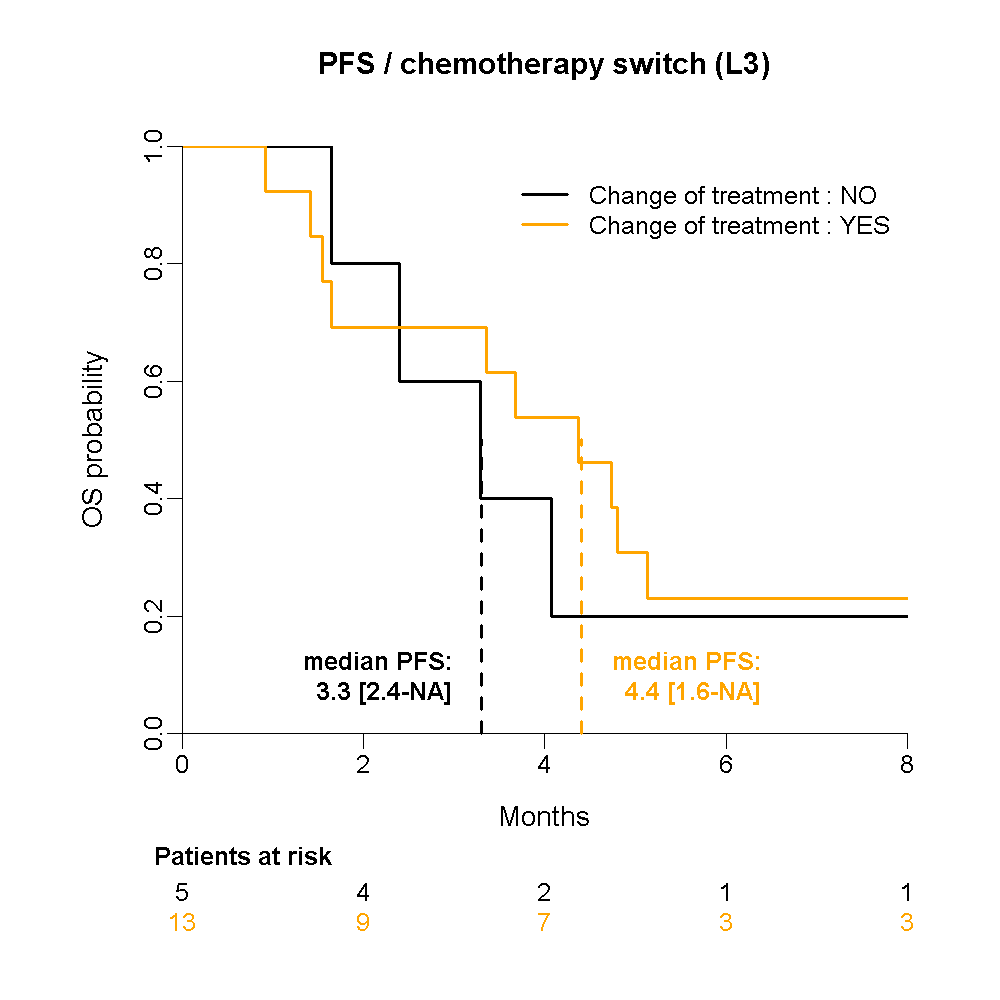

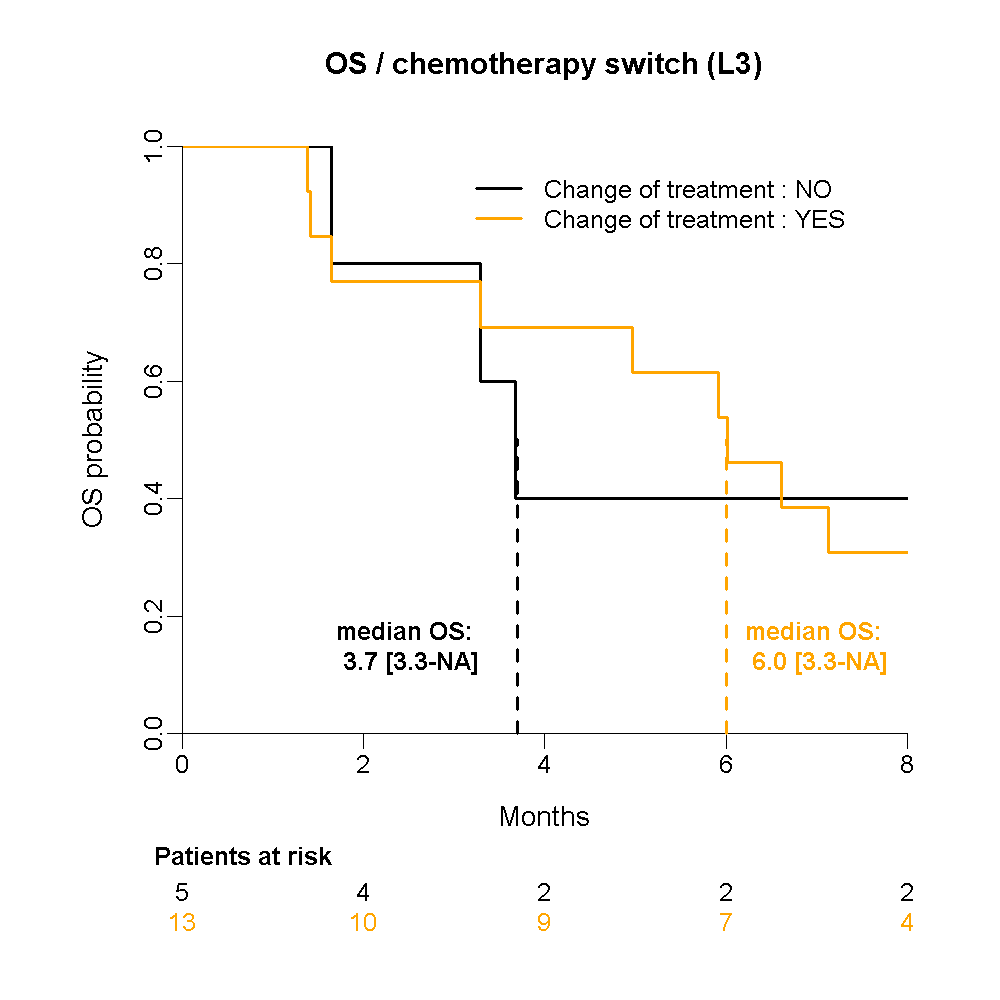


**A**

**B**


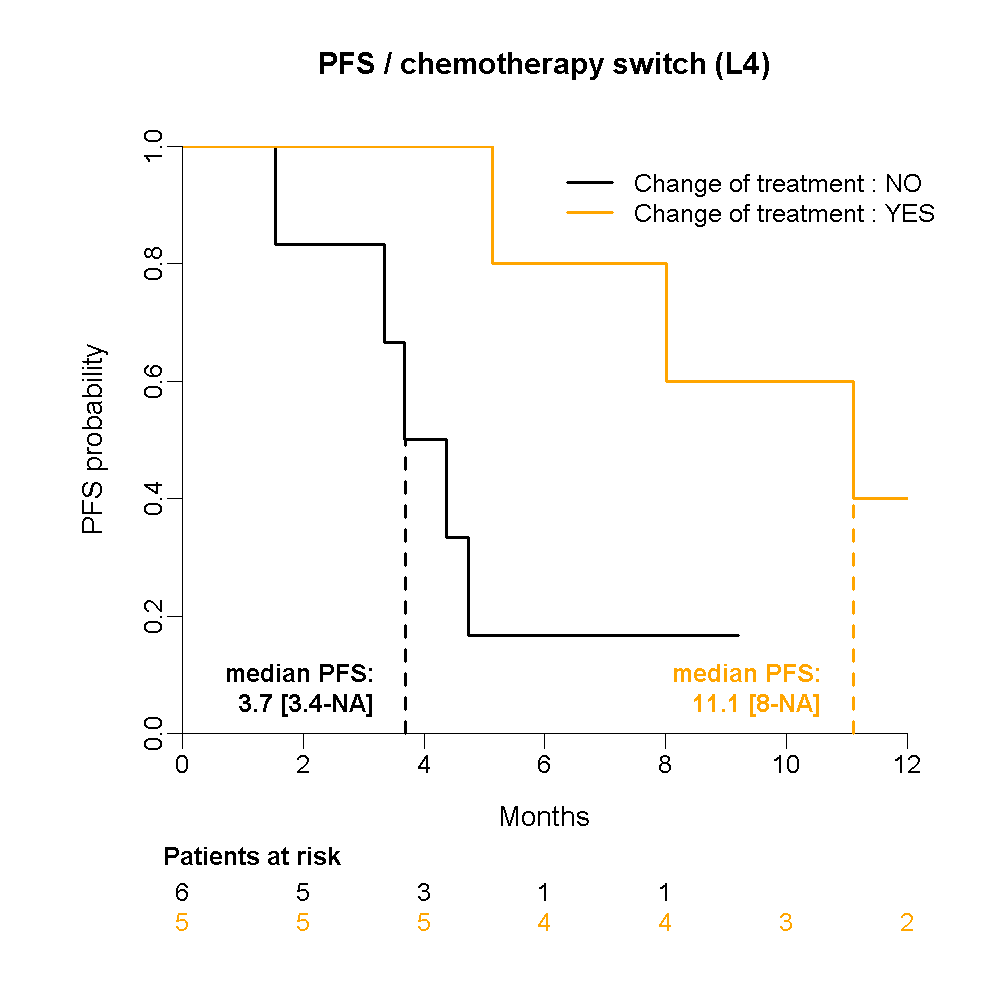

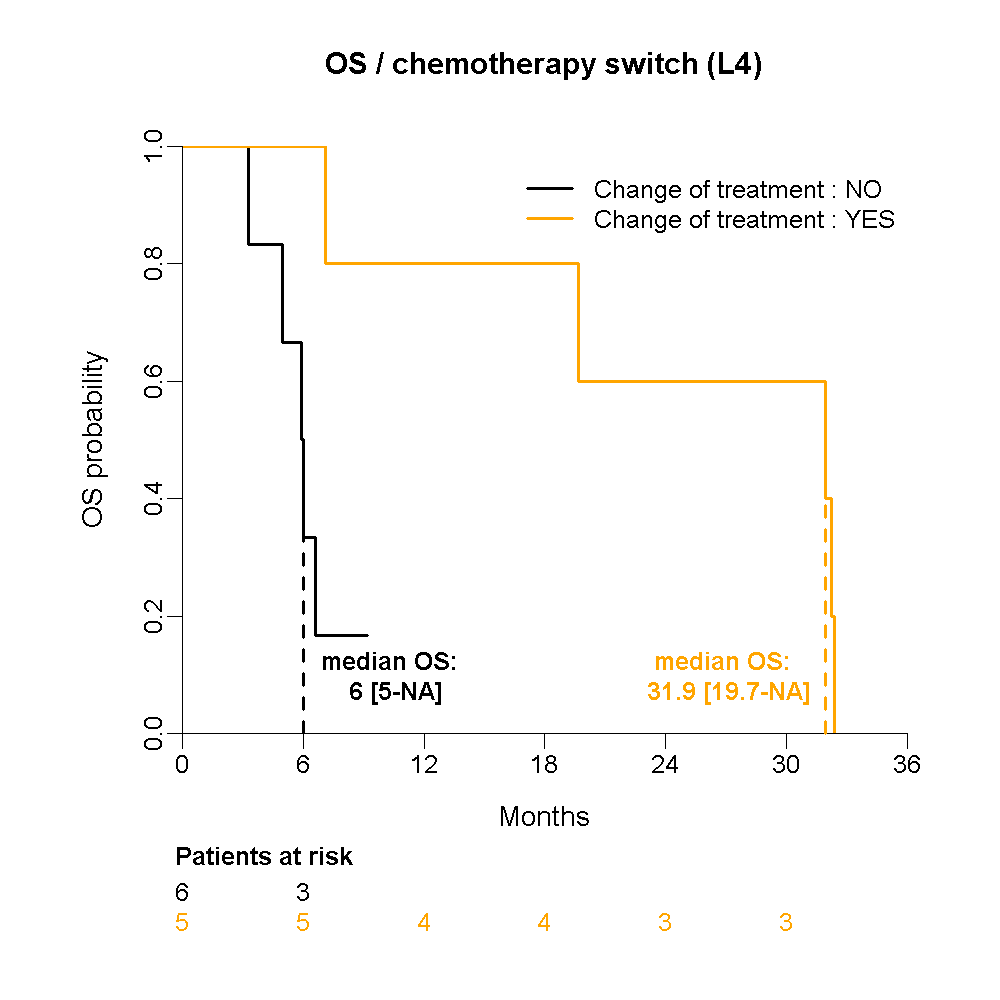


**D**

**C**

A PFS at 3rd line of chemotherapy

B OS at 3rd line of chemotherapy

C PFS at 4th line of chemotherapy

D OS at 4th line of chemotherapy
